# Synthesis and Biological Evaluation of Novel Dehydroabietic Acid Derivatives Conjugated with Acyl-Thiourea Peptide Moiety as Antitumor Agents

**DOI:** 10.3390/ijms160714571

**Published:** 2015-06-26

**Authors:** Le Jin, Hong-En Qu, Xiao-Chao Huang, Ying-Ming Pan, Dong Liang, Zhen-Feng Chen, Heng-Shan Wang, Ye Zhang

**Affiliations:** 1 State Key Laboratory Cultivation Base for the Chemistry and Molecular Engineering of Medicinal Resources, School of Chemistry & Pharmaceutical Science of Guangxi Normal University, Yucai Road 15, Guilin 541004, China; E-Mails: jindonhua@163.com (L.J.); quhongen-2003@163.com (H.-E.Q.); viphuangxc@126.com (X.-C.H.); panym2005@sina.com (Y.-M.P.); sky_8912@163.com (D.L.); chenzfgxnu@yahoo.com (Z.-F.C.); 2 Department of Chemistry & Pharmaceutical Science, Guilin Normal College, Xinyi Road 21, Guilin 541001, China

**Keywords:** dehydroabietic acid, chiral amino acid, thioureas, antitumor activity, apoptosis

## Abstract

A series of dehydroabietic acid (DHAA) acyl-thiourea derivatives were designed and synthesized as potent antitumor agents. The *in vitro* pharmacological screening results revealed that the target compounds exhibited potent cytotoxicity against HeLa, SK-OV-3 and MGC-803 tumor cell lines, while they showed lower cytotoxicity against HL-7702 normal human river cells. Compound **9n** (IC_50_ = 6.58 ± 1.11 μM) exhibited the best antitumor activity against the HeLa cell line and even displayed more potent inhibitory activity than commercial antitumor drug 5-FU (IC_50_ = 36.58 ± 1.55 μM). The mechanism of representative compound **9n** was then studied by acridine orange/ethidium bromide staining, Hoechst 33,258 staining, JC-1 mitochondrial membrane potential staining, TUNEL assay and flow cytometry, which illustrated that this compound could induce apoptosis in HeLa cells. Cell cycle analysis indicated that compound **9n** mainly arrested HeLa cells in the S phase stage. Further investigation demonstrated that compound **9n** induced apoptosis of HeLa cells through a mitochondrial pathway.

## 1. Introduction

As one of the main causes of mortality worldwide, cancer has became a global health problem. Thus, the design and synthesis of new antitumor drugs has attracted considerable efforts. Over the past decades, researchers have long explored natural products in the quest for new antitumor drugs. The success of doxorubicin, paclitaxel, and vinblastine has revealed that natural compounds are a rich source of antitumor drugs [1,2,3]. In fact, about 60% of antitumor agents derive from natural compounds [4].

As a natural occurring diterpene, rosin acid, DHAA and its derivatives display a fascinating spectrum of biological activities, such as anti-ulcer, antimicrobial, anxiolytic, antiviral and antitumor activities [5,6,7,8,9]. Recent reports have indicated that DHAA and their analogs have anticancer activity in many human cancer cells, including cervical carcinoma cells, hepatocellular carcinoma cells and breast cancer cells [10]. Our previous study has also demonstrated that the α-aminophosphonate, dipeptide, thiourea derivatives of DHAA showed potent antitumor activity [11,12,13]. So many DHAA derivatives containing functional groups have been designed and synthesized to screen for new potential antitumor agents [14,15,16].

**Scheme 1:**
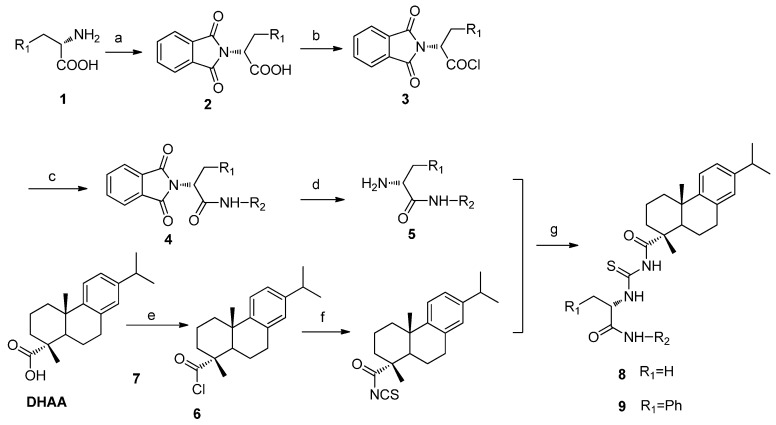
Synthetic pathway to target compounds **8a**–**8o** and **9a**–**9o**. Reagents and conditions: (**a**) phthalic anhydride, CH_3_COOH, 50 °C; (**b**) oxalyl chloride, CH_2_Cl_2_, r.t.; (**c**) aromatic primary amines, Et_3_N, CH_2_Cl_2_, r.t.; (**d**) hydrazine hydrate, CH_3_OH, r.t.; (**e**) oxalyl chloride, CH_2_Cl_2_, r.t.; (**f**) C_6_H_5_CH_3_, KSCN 110 °C; (**g**) CH_2_Cl_2_, r.t.

Continuing our research program on the synthesis of DHAA derivatives as potent antitumor agents, the present work in this paper is to design and synthesize a series of new DHAA acyl-thiourea derivatives by the introduction of acyl-thiourea group in the carboxylic acid group of DHAA (Scheme 1). Many acyl-thiourea derivatives exhibit good inhibitory activity against malignant tumors [17], and our previous work has also demonstrated that the introductions of α-aminophosphonate, dipeptide, and thiourea on DHAA could lead to improved antitumor activity. Thus, it is reasonable to consider that well-designed functional groups would enable a fine-tuning of special properties of a pharmacy core, and to expect that the introduction of an acyl-thiourea moiety on a DHAA skeleton may lead to good antitumor activity. Therefore, the synthetic route to DHAA acyl-thiourea derivatives was herein designed and carried out. Also, the cytotoxicity *in vitro* against the HeLa, SK-OV-3 and MGC-803 tumor cell lines and HL-7702 normal human river cell line was evaluated. Furthermore, the molecules mechanism of apoptotic pathway induced apoptosis in HeLa cells by the representative compound of the target compound was also investigated.

## 2. Results and Discussion

### 2.1. Chemistry

DHAA acyl-thiourea derivatives were synthesized as outlined in Scheme 1. Compound **2** was prepared by the condensation of l-amino acid **1** with phthalic anhydride in the presence of acetic acid. Compound **3** was obtained by the treatment of compound **2** and oxalyl chloride, and it was then treated with series of aromatic primary amines to offer compounds **4**. Compounds **5** were synthesized by the treatment of compounds **4** with hydrazine hydrate in the presence of ethanol at room temperature. Meanwhile DHAA was treated with oxalyl chloride to offer compound **6**. Then compound **6** was treated with KSCN to offer compound **7**. Compounds **8** and **9** were finally acquired by the condensation of compound **7** and compounds **5** in CH_2_Cl_2_ at room temperature. The structures of DHAA acyl-thiourea derivatives **8**–**9** were confirmed by ^1^H NMR, ^13^C NMR and high-resolution mass spectroscopy.

### 2.2. Biological Activity

#### 2.2.1. MTT Assay

The *in vitro* cytotoxic potency of DHAA acyl-thiourea derivatives **8a**–**8o** and **9a**–**9o** were evaluated by 3-(4,5-dimethylthiazol-2-yl)-2,5-diphenyltetrazolium bromide (MTT) assay against HeLa, SK-OV-3 and MGC-803 tumor cell lines, with 5-FU as the positive control. The tested results were shown in Table 1.

**Table 1:** Effect of compounds **8a**–**8o** and **9a**–**9o** against cell viability of different cell lines.

Compound	IC_50_ (μM·L^−1^)					
	R_1_	R_2_	HeLa	SK-OV-3	MGC-803	HL-7702
**8a**	H	Ph	31.09 ± 2.13	26.49 ± 2.53	28.49 ± 1.79	47.59 ± 2.43
**8b**	H	*o*-Ph-CH_3_	29.19 ± 2.43	42.81 ± 1.42	19.17 ± 1.66	52.51 ± 1.25
**8c**	H	*p*-Ph-CH_3_	38.71 ± 3.65	50.87 ± 4.11	24.85 ± 1.64	61.67 ± 3.41
**8d**	H	*o*-Ph-Cl	19.73 ± 1.23	48.33 ± 4.21	20.55 ± 1.57	69.43 ± 4.23
**8e**	H	*p*-Ph-Cl	36.15 ± 3.31	36.11 ± 3.28	21.73 ± 1.69	57.67 ± 3.66
**8f**	H	*o*-Ph-OCH_3_	28.08 ± 2.11	23.03 ± 3.01	17.95 ± 1.73	44.73 ± 3.34
**8g**	H	*p*-Ph-OCH_3_	27.42 ± 2.43	26.81 ± 2.47	18.56 ± 1.72	43.61 ± 2.43
**8h**	H	*o*-Ph-F	23.27 ± 1.55	31.81 ± 2.36	26.06 ± 2.37	53.52 ± 2.35
**8i**	H	*p*-Ph-F	30.24 ± 2.55	32.48 ± 2.64	29.42 ± 2.77	54.45 ± 2.34
**8j**	H	*o*-Ph-Br	22.75 ± 1.55	39.08 ± 3.56	27.93 ± 3.26	59.57 ± 3.54
**8k**	H	*p*-Ph-Br	19.03 ± 1.46	40.77 ± 3.57	29.98 ± 1.24	54.65 ± 3.65
**8l**	H	3,5-di-methyl-Ph	18.64 ± 1.24	32.97 ± 2.59	13.88 ± 1.32	55.29 ± 2.65
**8m**	H	1-Naphthyl	46.55 ± 4.77	49.42 ± 4.64	36.48 ± 3.24	59.45 ± 4.65
**8n**	H	2-pyridyl	14.32 ± 1.33	25.23 ± 2.43	19.33 ± 1.32	48.75 ± 2.44
**8o**	H	3,4,5-tri-methoxyl-Ph	18.06 ± 1.23	29.56 ± 2.67	22.67 ± 1.77	49.22 ± 2.65
**9a**	Ph	Ph	25.68 ± 2.23	17.01 ± 1.13	26.64 ± 3.25	37.45 ± 1.44
**9b**	Ph	*o*-Ph-CH_3_	17.66 ± 1.34	18.49 ± 1.44	14.14 ± 1.12	38.56 ± 1.65
**9c**	Ph	*p*-Ph-CH_3_	19.08 ± 1.08	16.21 ± 1.98	19.18 ± 1.66	36.24 ± 1.39
**9d**	Ph	*o*-Ph-Cl	16.99 ± 1.76	16.93 ± 1.43	20.35 ± 3.41	36.53 ± 1.43
**9e**	Ph	*p*-Ph-Cl	17.21 ± 1.25	18.47 ± 1.27	17.08 ± 2.87	38.49 ± 1.22
**9f**	Ph	*o*-Ph-OCH_3_	28.06 ± 2.23	18.91 ± 1.72	20.66 ± 1.41	38.44 ± 1.45
**9g**	Ph	*p*-Ph-OCH_3_	15.58 ± 1.53	20.10 ± 1.78	26.13 ± 2.63	41.15 ± 1.32
**9h**	Ph	*o*-Ph-F	18.83 ± 1.45	28.79 ± 2.32	24.67 ± 3.33	49.39 ± 2.64
**9i**	Ph	*p*-Ph-F	23.71 ± 1.01	26.47 ± 2.07	21.40 ± 1.88	46.44 ± 2.44
**9j**	Ph	*o*-Ph-Br	12.66 ± 1.03	40.27 ± 3.41	21.33 ± 1.27	60.47 ± 3.43
**9k**	Ph	*p*-Ph-Br	15.66 ± 1.13	46.69 ± 4.76	25.51 ± 2.47	66.49 ± 4.48
**9l**	Ph	3,5-di-methyl-Ph	15.81 ± 1.55	31.52 ± 2.23	18.05 ± 152	53.42 ± 2.24
**9m**	Ph	1-Naphthyl	12.75 ± 1.20	15.29 ± 1.73	22.87 ± 1.66	35.59 ± 1.53
**9n**	Ph	2-pyridyl	6.58 ± 1.11	19.61 ± 1.87	16.90 ± 1.56	39.61 ± 1.47
**9o**	Ph	3,4,5-tri-methoxyl-Ph	17.51 ± 1.54	29.30 ± 2.04	29.32 ± 2.09	59.34 ± 2.54
**DHA**			30.35 ± 3.45	87.52 ± 4.12	>100	NT ^a^
**5-FU**			36.58 ± 1.55	30.25 ± 1.13	34.85 ± 1.75	NT ^a^

^a^ IC_50_ values are presented as the mean ± SD (standard error of the mean) from three independent experiments.

As can be seen from the Table 1, most target compounds showed certain anticancer activities against the tumor cells (HeLa, SK-OV-3, and MGC-803) as compared with the control 5-fluorouracil (5-FU). Compound **9n** (IC_50_ = 6.58 ± 1.11 μM) exhibited the best antitumor activity against the HeLa cell line and even displayed more potent inhibitory activity than commercial antitumor 5-FU (IC_50_ = 36.58 ± 1.55 μM). All the compounds showed lower cytotoxicity on HL-7702 cells than on that of these three cancer cell lines.

From the above results, some interesting structure-activity relationships could be concluded: (1) the introduction of acyl-thiourea was significant for improving their activity; (2) in HeLa, SK-OV-3 and MGC-803 assays, the antitumor activities were found to be in the order of ortho- > para-; (3) compared the antitumor activity of compounds **8** with **9**, it could be found that the antitumor activity of compounds **9** were better than that of **8**. It was important to note that the introduction of a benzene group at R_1_ was important for improving antitumor activities.

#### 2.2.2. Apoptosis Assessment by AO/EB Staining

**Figure 1:**
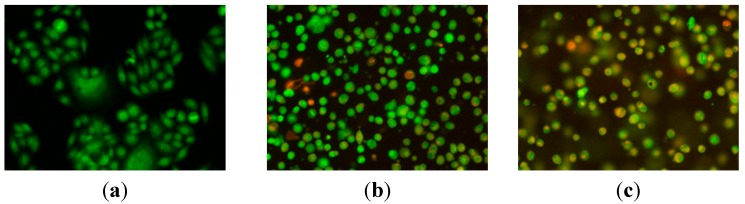
AO/EB staining of compound **9n** in HeLa cells. (**a**) Not treated with the **9n** were used as control at for 24 h and (**b**,**c**) treatment with compound **9n** (10 μM) for 12 and 24 h, respectively.

The cytotoxicity of compound **9n** at a concentration of 10 μM against HeLa cells from 12 to 24 h was detected by AO/EB staining, and Hela cells not treated with the **9n** were used as control for 48 h. The results are shown in Figure 1. Results depicted in Figure 1 indicate that control cells did not take up EB and appeared faint orange-red, while cells treated with **9n** at 10 μM showed obvious apoptotic characters (chromatin condensation or fragmentation) and appeared intense orange-red, as dead cells had ruptured membranes, which allowed EB to enter into the cells. Also due to the AO uptake, control cells appeared green while **9n** treated cells appeared green to intense green as apoptotic cells had much more permeable membranes. These findings indicated that compound **9n** was able to induce apoptosis.

#### 2.2.3. Apoptosis Assessment by Hoechst 33258 Staining

**Figure 2:**
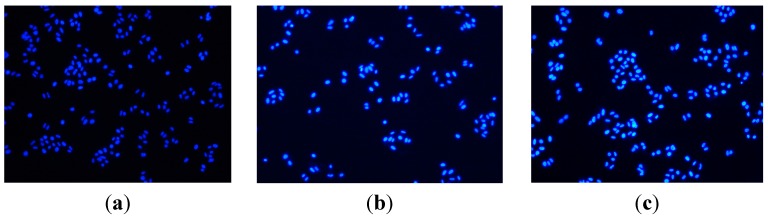
Hoechst 33,258 staining of compound **9n** in HeLa cells. (**a**) Cells not treated with compound **9n** were used as control for 24 h and (**b**,**c**) treatment with compound **9n** (10 μM) for 12 and 24 h, respectively.

Hoechst 33258 which stains the cell nucleus, is a membrane permeable dye with blue fluorescence. Live cells with uniformly light blue nuclei were obviously detected under the fluorescence microscope after treatment with Hoechst 33258 whereas apoptotic cells had bright blue nuclei due to karyopyknosis and chromatin condensation. However, the nuclei of dead cells could not be stained. HeLa cells treated with compound **9n** at 10 μM from 12 to 24 h were stained with Hoechst 33258. HeLa cells not treated with compound **9n** were used as control for 24 h. The results are shown in Figure 2. As shown in Figure 2, HeLa cells not treated with compound **9n** were normally blue (in the web version). It is worth noting that, for **9n** treatment, the cells displayed strong blue fluorescence and indicated typical apoptotic morphology after 12 and 24 h. The observation revealed that compounds **9n** induced apoptosis in HeLa cells.

#### 2.2.4. Mitochondrial Membrane Potential Staining

**Figure 3:**
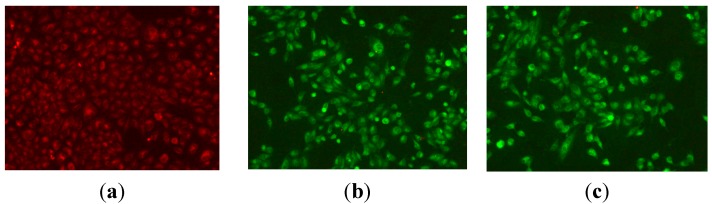
JC-1 mitochondrial membrane potential staining of compound **9n** in HeLa cells. (**a**) Cells not treated with the **9n** were used as control at for 24 h and (**b**,**c**) treatment with compound **9n** (10 μM) for 12 and 24 h, respectively.

Apoptosis plays an important role in cancer, since its induction in tumor cells is essential for successful treatment [18]. Mitochondria play key roles in apoptosis through the release of pro-apoptotic factors such as cytochrome *c* and apoptosis-inducing factor [19,20]. In order to further investigate the apoptosis-inducing effect of target compound **9n**, mitochondrial membrane potential changes were designed and detected, using the fluorescent probe JC-1. JC-1 exhibited potential dependent accumulation in mitochondria, indicated by a fluorescence emission shift from red (~590 nm) to green (~545 nm) [21]. In the control cells, JC-1 could aggregate in mitochondria and present high red fluorescence. However, in cells undergoing apoptosis, where the mitochondrial potential has collapsed, JC-1 exists in the cytosol as a monomer that emits green fluorescence. HeLa cells treated with compound **9n** at 10 μM from 12 to 24 h were stained with JC-1 and cells not treated with the compound **9n** were used as control for 24 h. The results are shown in Figure 3. The JC-1 monomer and J-aggregates were excited at 514 and 585 nm, respectively, and light emissions were collected at 515–545 nm (green) and 570–600 nm (red). For fluorescence microscopy, Figure 3 showed that cells not treated with the compound **9n** were normally red (in the web version), while for compound **9n** treatment, cells showed strong green fluorescence and indicated typical apoptotic morphology after 12 and 24 h. Therefore, it could be concluded that compound **9n** induced apoptosis against HeLa cell line. The results were identical with that of previous experiment of Hoechst 33,258 staining.

#### 2.2.5. TUNEL Assay

**Figure 4:**
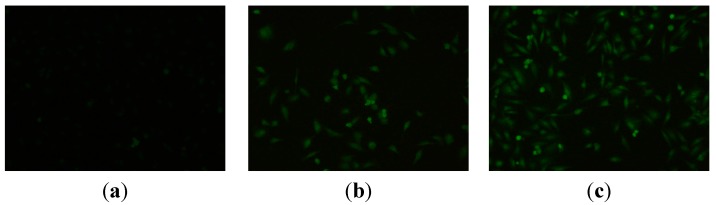
TUNEL assay of compound **9n** in HeLa cells. (**a**) Cells not treated with the **9n** were used as control for 24 h and (**b**,**c**) treatment with compound **9n** (10 μM) for 12 and 24 h, respectively.

To further define the mechanism of cell death caused by compound **9n**, HeLa cells were treated with compound **9n** at 10 μM from 12 to 24 h and the DNA fragmentation was measured by the TUNEL assay. TUNEL (terminal-deoxynucleotidyl transferase meditated nick end labeling) is a common method for identifying apoptotic cells *in situ* by detecting DNA fragmentation. When the genomic DNA is broken, the exposed 3ʹ-OH at the end of deoxynucleotide transfer as the catalytic plus green fluorescent probes fluorescein (FITC) labeled dUTP, which can be detected by fluorescence microscopy or flow cytometry, as indicated by a green color (in the web version). The results are illustrated in Figure 4. For fluorescence microscopy, Figure 4 shows that HeLa cells treated with the compound **9n** at different time appeared in green (in the web version), exhibiting that compound **9n** significantly induced apoptosis against the HeLa cell line. The results were in good agreement with the previous experiments.

#### 2.2.6. Apoptosis Study by Flow Cytometry Assay

**Figure 5:**
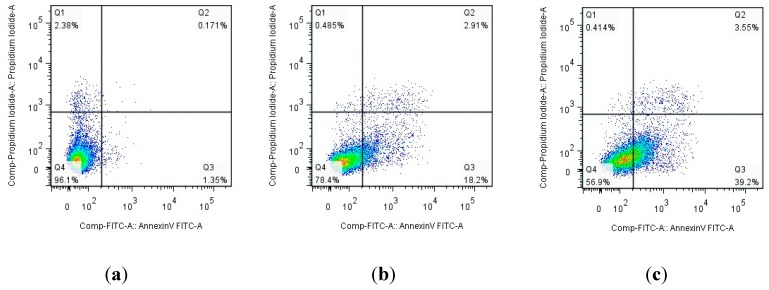
Apoptosis ratio detection of compound **9n** by Annexin V/PI assay. (**a**) Cells not treated with compund **9n** were used as control for 12 h; (**b**,**c**) HeLa cells were treated with compound **9n** at 10 and 15 μM for 12 h, respectively.

Annexin V/PI staining was performed to distinguish apoptotic and necrotic cell deaths. The apoptosis ratios induced by compound **9n** in HeLa cells were quantitatively determined by flow cytometry (Figure 5). Four quadrant images were observed by flow cytometric analysis: the Q1 area represented damaged cells appearing in the process of cell collection, the Q2 region showed necrotic cells and later period apoptotic cells; the early apoptotic cells were located in the Q3 area and the Q4 area showed normal cells. Figure 5 revealed that compound **9n** could induce apoptosis in HeLa cells. Apoptosis ratios (including the early and late apoptosis ratios) for compound **9n** were obtained after 12 h of treatment at the concentration of 10 and 15 μM. The apoptosis of HeLa cells treated with target compound **9n** increased gradually in a concentration manner. The apoptosis ratios of compound **9n** measured at different concentration points were found to be 21.11% and 42.75%, respectively, while that of the control was 1.521%. The results illustrated that target compound **9n** suppressed cell proliferation by inducing apoptosis.

#### 2.2.7. Investigation of Cell Cycle Distribution

**Figure 6:**
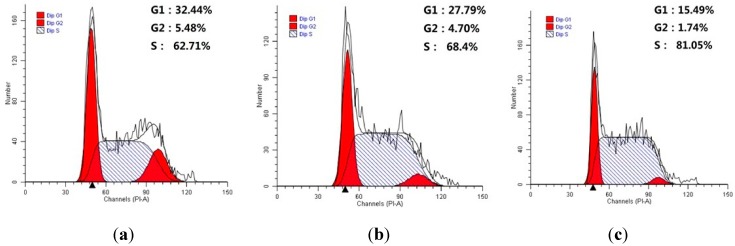
Investigation of cell cycle distribution by flow cytometric analysis. (**a**) Untreated HeLa cells as a control; (**b**,**c**) Cells treated with increasing concentrations of compound **9n** (10, 15 μM) for 48 h.

Many anticancer drugs interact with cells leading to cell growth arrest. To determine the possible role of cell cycle arrest in DHAA derivative induced growth inhibition, HeLa cells were treated with different concentrations of compound **9n**. Cell cycle distribution was investigated by flow cytometric analysis after staining of the DNA with propidium iodide (PI). After treatment with compound **9n** at different concentrations for 48 h, it was observed that the cells accumulated in G1 and G2 phase were gradually decreased, while S period cells compared with the control cells were gradually increased (Figure 6). These results suggested that target compound **9n** mainly arrested HeLa cells in the S stage.

#### 2.2.8. ROS Generation Assay and Intracellular Ca^2+^ Release

**Figure 7:**
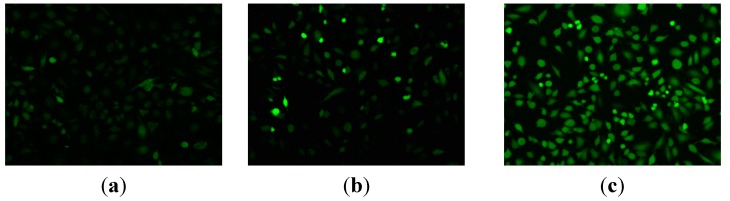
ROS generation assay of compound **9n** in HeLa cells. (**a**) Cells not treated with compound **9n** were used as control for 12 h and (**b**,**c**) treatment with compound **9n** (10, 15 μM) for 12 h, respectively.

**Figure 8:**
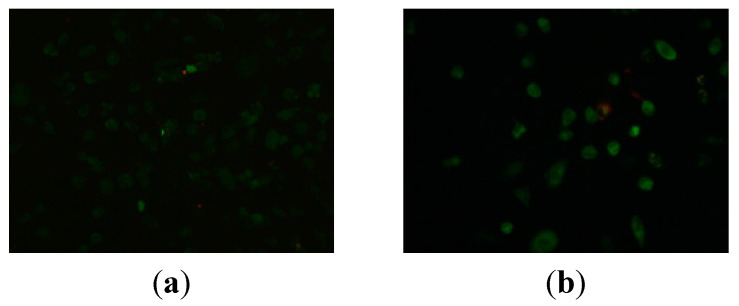
Investigation of the effect of compound **9n** on calcium release. (**a**) Cells not treated with the **9n** were used as control for 24 h; (**b**) HeLa cells treated with compound **9n** for 24 h at 10 μM, respectively.

Recently, oxidative damage to the mitochondrial membrane due to increased generation of reactive oxygen species (ROS) has been shown to play an important role in apoptosis [22,23,24]. Mitochondria have also been implicated as a source of ROS during apoptosis. In addition, a decrease of mitochondria membrane potential has recently been shown to lead to increased generation of ROS and apoptosis [25]. We herein studied whether the loss of mitochondrial transmembrane potential resulting in the generation of ROS measured as described in our previous papers , using the fluorescent probe 2,7-dichlorofluorescein diacetate (DCF-DA) determined by fluorescence microscopy. HeLa cells treated with compound **9n** displayed stronger fluorescence intensity in cytoplasm, while HeLa cells not treated with compound **9n** under the same experimental procedures were used as control and the fluorescence detected in these cells was weak and spread all over the cells. Fluorescence microscopy revealed that compound **9n** induced an increase of the ROS level in HeLa cells, as seen in Figure 7, indicating that compound **9n** significantly induced apoptosis in HeLa cells. Calcium has long been recognized as a significant participant in cell apoptosis. Therefore, we investigated whether compound **9n** inducing cell death was involved in the production of Ca^2+^. To investigate the effect of compound **9n** on calcium release, HeLa cells treated with compound **9n** at 10 μM for 24 h were loaded with the Ca^2+^ indicator Fluo-3-acetoxymethyl-ester (Fluo-3/AM, 5 µM; Beyotime, Jiangsu, China). Figure 8 shows that HeLa cells treated with compound **9n** displayed stronger green fluorescence, while HeLa cells not treated with compound **9n** under the same experimental procedures were used as control and the fluorescence detected in these cells were exhibited normally as green (in the web version). These results imply that the increment of ROS and intracellular Ca^2+^ released might play a role as an early mediator in compound **9n** induced apoptosis.

#### 2.2.9. Caspase-3 Activation Assay

**Figure 9:**
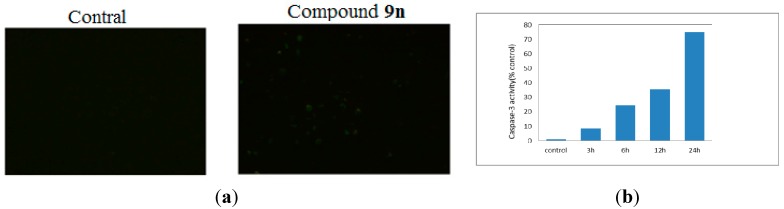
(**a**) Activation of Caspase-3 induced by compound **9n**, examined by FITC-DEVD-FMK under a fluorescent microscope; (**b**) Measurement of caspase-3 activity. Dose-dependent induction of caspase-3 in human HeLa cell line.

It is well known that an increase of intracellular ROS can lead to apoptosis. To explore the signaling pathway involved in compound **9n** induced apoptosis in HeLa cells, a decrease of mitochondria membrane potential lead to increased generation of ROS was investigated, which was known to be frequently involved in a chemically induced apoptotic signaling pathway and the resultant activation of caspase cascade [26,27]. Caspases are a family of cysteinyl aspartate specific proteases involved in apoptosis and are dichotomized to groups of initiators (caspases 8, 9 and 10) and executioners (caspases 3, 6 and 7) [28,29]. Caspase-3 is able to directly degrade multiple substrates including structural and regulatory proteins. Some small molecules have been developed to be selective activators of Caspase-3 [30]. Thus, we investigated whether caspase-3 was activated when HeLa cells were exposed to compound **9n**. As shown in Figure 9a, cells treated with compound **9n** had a significant increase in the activity of caspase-3 as indicated by a notable shift in the ratio of green/dark fluorescence *versus* control. As shown in Figure 9b, the results clearly showed that an induction of cell apoptosis took place when the cells were treated with compound **9n**, and the stimulation of caspase-3 activity both increased in a time dependent manner from 3 to 24 h.

## 3. Experimental Section

### 3.1. Chemistry

Experimental: NMR spectra were recorded on a BRUKER AVANCE 400 NMR spectrometer (Bruker, Rheinstetten, Germany) in CDCl_3_. Mass spectra were determined on a FTMS ESI spectrometer (Thermo, Waltham, MA, USA).

General procedure for the preparation of compounds 8 and 9: l-amino acid (1 mmol) and phthalic anhydride (1.2 mmol) were added to CH_3_COOH (25 mL) and the mixture was stirred at 50 °C for 12 h to offer compound **2**. Compound **2** (1 mmol) added to dry CH_2_Cl_2_ (15 mL) was stirred at 0 °C and oxalyl chloride (1.5 mmol) was dripped into the mixture and stirred at room temperature for 6 h. After the reaction, the solvent and excess oxalyl chloride was evaporated under reduced pressure. Aromatic primary amines (1 mmol) and triethylamine (0.5 mmol) were added to the mixture and stirred at room temperature for 0.5 h. After the reaction, the solvent was evaporated under reduced pressure, and the crude product was purified by chromatography on silica gel eluted with petroleum ether/ethyl acetate (*V:V* = 6:1) to offer compound **4**. Compound **4** (1 mmol) and hydrazine hydrate (3 mmol) were added to ethanol (15 mL) and the mixture was stirred at room temperature for 8 h. After the reaction was completed, the solvent was evaporated under reduced pressure, and the crude product was purified by chromatography on silica gel eluted with petroleum ether/ethyl acetate (*V:V* = 3:1) to obtain compounds **5**. DHAA (1 mmol) added to dry CH_2_Cl_2_ (15 mL) was stirred at 0 °C and oxalyl chloride (1.5 mmol) was dripped into the mixture and stirred at room temperature for 6 h. After the reaction, the solvent and excess oxalyl chloride was evaporated under reduced pressure to offer compound **6**. Compound **6** (1 mmol) and KSCN (1.2 mmol) were added to toluene (15 mL) and the mixture was stirred at 110 °C for 12 h to offer compound **7**. Compounds **5** (1 mmol) and compound **7** (1 mmol) were added to CH_2_Cl_2_ (15 mL) and the mixture was stirred at room temperature for 0.5 h. After the reaction, the solvent was evaporated under reduced pressure, and the crude product was purified by chromatography on silica gel eluted with petroleum ether/ethyl acetate (*V:V* = 6:1) to offer compounds **8a**–**8o** and **9a**–**9o**.

*(1R,4aS)-7-Isopropyl-1,4a-dimethyl-N-(((R)-1-oxo-1-(phenylamino)propan-2-yl)carbamothioyl)-1,2,3,4,4a,9,10,10a-octahydrophenanthrene-1-carboxamide* (**8a**). Yields 85.52%; ^1^H NMR (400 MHz, CDCl_3_): δ 11.13 (d, *J* = 7.5 Hz, 1H, NH), 8.71 (s, 1H, NH), 8.61 (s, 1H, NH), 7.58 (d, *J* = 7.6 Hz, 2H), 7.34 (t, *J* = 8.0 Hz, 2H), 7.18 (d, *J* = 8.2 Hz, 1H), 7.13 (s, 1H), 7.04 (d, *J* = 6.4 Hz, 1H), 6.91 (s, 1H), 5.16–5.10 (m, 1H), 2.95–2.80 (m, 3H), 2.36 (d, *J* = 13.3 Hz, 1H), 2.10 (d, *J* = 12.5 Hz, 1H), 1.80–1.69 (m, 5H), 1.61 (d, *J* = 6.9 Hz, 3H, CH_3_), 1.53–1.46 (m, 2H), 1.36 (s, 3H, CH_3_), 1.25 (s, 6H, 2 × CH_3_), 1.23 (s, 3H, CH_3_). ^13^C NMR (100 MHz, CDCl_3_): δ 180.1, 179.5, 168.5, 146.1, 146.1, 137.6, 134.2, 129.0, 126.9, 124.4, 124.1, 123.9, 119.9, 55.2, 48.6, 45.2, 37.6, 37.1, 37.0, 33.4, 29.7, 25.1, 23.9, 21.4, 18.4, 16.7, 16.1. HR-MS (*m/z*) (ESI): calcd for C_30_H_39_N_3_O_2_S [M − H^+^]: 504.26847; found: 504.26982.

*(1R,4aS)-7-Isopropyl-1,4a-dimethyl-N-(((R)-1-oxo-1-(o-tolylamino)propan-2-yl)carbamothioyl)-1,2,3,4,4a,9,10,10a-octahydrophenanthrene-1-carboxamide* (**8b**). Yields 84.32%; ^1^H NMR (400 MHz, CDCl_3_): δ 11.10 (d, *J* = 7.7 Hz, 1H, NH), 8.63 (s, 1H, NH), 8.22 (s, 1H, NH), 7.87 (d, *J* = 7.7 Hz, 1H), 7.21 (d, *J* = 8.4 Hz, 1H), 7.06 (d, *J* = 8.2 Hz, 1H), 7.00 (d, *J* = 8.0 Hz, 2H), 6.88 (s, 1H), 6.71–6.65 (m, 1H), 5.17–5.13 (m, 1H), 2.93–2.77 (m, 3H), 2.32 (d, *J* = 12.9 Hz, 1H), 2.27 (s, 3H, CH_3_), 2.07 (d, *J* = 10.7 Hz, 1H), 1.80–1.66 (m, 5H), 1.59 (d, *J* = 6.9 Hz, 3H, CH_3_), 1.50–1.42 (m, 2H), 1.32 (s, 3H, CH_3_), 1.22 (d, *J* = 2.8 Hz, 6H, 2 × CH_3_), 1.21 (s, 3H, CH_3_). ^13^C NMR (100 MHz, CDCl_3_): δ 179.8, 179.4, 168.6, 146.1, 145.9, 135.5, 134.1, 130.4, 128.8, 126.8, 126.5, 124.0, 122.6, 121.2, 118.4, 55.1, 48.5, 45.1, 37.5, 36.9, 36.9, 33.3, 29.6, 25.0, 23.9, 21.3, 18.1, 17.3, 16.8, 16.0. HR-MS (*m/z*) (ESI): calcd for C_31_H_41_N_3_O_2_S [M − H^+^]: 518.28412; found: 518.28514.

*(1R,4aS)-7-Isopropyl-1,4a-dimethyl-N-(((R)-1-oxo-1-(p-tolylamino)propan-2-yl)carbamothioyl)-1,2,3,4,4a,9,10,10a-octahydrophenanthrene-1-carboxamide* (**8c**). Yields 81.26%; ^1^H NMR (400 MHz, CDCl_3_): δ 11.11 (d, *J* = 7.5 Hz, 1H, NH), 8.59 (d, *J* = 4.3 Hz, 2H, NH), 7.43 (d, *J* = 8.4 Hz, 2H), 7.15 (d, *J* = 8.2 Hz, 1H), 7.10 (d, *J* = 8.2 Hz, 2H), 7.01 (dd, *J* = 8.2, 1.7 Hz, 1H), 6.88 (d, *J* = 1.3 Hz, 1H), 5.08 (dd, *J* = 13.5, 7.3 Hz, 1H), 3.00–2.74 (m, 3H), 2.33 (d, *J* = 12.3 Hz, 1H), 2.30 (s, 3H, CH_3_), 2.07 (d, *J* = 10.6 Hz, 1H), 1.79–1.64 (m, 5H), 1.58 (d, *J* = 6.9 Hz, 3H, CH_3_), 1.51–1.43 (m, 2H), 1.33 (s, 3H, CH_3_), 1.23 (s, 6H, 2 × CH_3_), 1.21 (s, 3H, CH_3_). ^13^C NMR (100 MHz, CDCl_3_): δ 179.8, 179.4, 168.4, 146.1, 146.0, 135.0, 134.2, 134.0, 129.4, 126.9, 124.0, 123.9, 119.9, 55.2, 48.6, 45.1, 37.5, 37.0, 37.0, 33.4, 29.6, 25.1, 23.9, 21.4, 20.8, 18.4, 16.8, 16.0. HR-MS (*m/z*) (ESI): calcd for C_31_H_41_N_3_O_2_S [M − H^+^]: 518.28412; found: 518.28516.

*(1R,4aS)-N-(((R)-1-((2-Chlorophenyl)amino)-1-oxopropan-2-yl)carbamothioyl)-7-isopropyl-1,4a-dimethyl-1,2,3,4,4a,9,10,10a-octahydrophenanthrene-1-carboxamide* (**8d**). Yields 84.16%; ^1^H NMR (400 MHz, CDCl_3_): δ 11.15 (d, *J* = 7.4 Hz, 1H, NH), 8.67 (s, 1H, NH), 8.51 (s, 1H, NH), 8.36 (d, *J* = 8.2 Hz, 1H), 7.36 (dd, *J* = 8.0, 1.4 Hz, 1H), 7.26 (d, *J* = 7.2 Hz, 1H), 7.15 (d, *J* = 8.2 Hz, 1H), 7.05 (d, *J* = 9.1 Hz, 1H), 7.01 (d, *J* = 8.3 Hz, 1H), 6.89 (s, 1H), 5.20–5.14 (m, 1H), 2.92–2.79 (m, 3H), 2.33 (d, *J* = 13.3 Hz, 1H), 2.09 (d, *J* = 12.4 Hz, 1H), 1.83–1.68 (m, 5H), 1.62 (d, *J* = 7.0 Hz, 3H, CH_3_), 1.52–1.46 (m, 2H), 1.34 (s, 3H, CH_3_), 1.23 (s, 6H, 2 × CH_3_), 1.21 (s, 3H, CH_3_). ^13^C NMR (100 MHz, CDCl_3_): δ 180.0, 179.4, 168.7, 146.0, 145.9, 134.1, 134.1, 129.0, 127.5, 126.8, 124.9, 123.9, 123.9, 123.1, 121.7, 55.4, 48.5, 45.1, 37.5, 36.9, 36.8, 33.3, 29.6, 25.0, 23.9, 21.3, 18.3, 17.1, 16.0. HR-MS (*m/z*) (ESI): calcd for C_30_H_38_ClN_3_O_3_S [M − H^+^]: 538.22950; found: 538.23111.

*(1R,4aS)-N-(((R)-1-((4-Chlorophenyl)amino)-1-oxopropan-2-yl)carbamothioyl)-7-isopropyl-1,4a-dimethyl-1,2,3,4,4a,9,10,10a-octahydrophenanthrene-1-carboxamide* (**8e**). Yields 84.78%; ^1^H NMR (400 MHz, CDCl_3_): δ 11.10 (d, *J* = 7.5 Hz, 1H, NH), 8.86 (s, 1H, NH), 8.60 (s, 1H, NH), 7.51 (d, *J* = 8.9 Hz, 2H), 7.29–7.26 (m, 2H), 7.15 (s, 1H), 7.02 (dd, *J* = 8.2, 1.7 Hz, 1H), 6.89 (d, *J* = 1.4 Hz, 1H), 5.11–5.07 (m, 1H), 2.93–2.80 (m, 3H), 2.34 (d, *J* = 13.3 Hz, 1H), 2.07 (d, *J* = 12.0 Hz, 1H), 1.82–1.69 (m, 5H), 1.58 (d, *J* = 6.9 Hz, 3H, CH_3_), 1.51–1.44 (m, 2H), 1.34 (s, 3H, CH_3_), 1.23 (s, 6H, 2 × CH_3_), 1.21 (s, 3H, CH_3_). ^13^C NMR (100 MHz, CDCl_3_): δ 180.2, 179.6, 168.49, 146.1, 146.1, 136.2, 134.1, 129.3, 129.0, 126.9, 124.1, 123.9, 121.2, 55.1, 48.7, 45.2, 37.6, 37.1, 37.0, 33.4, 29.7, 25.1, 23.9, 21.5, 18.4, 16.5, 16.1. HR-MS (*m/z*) (ESI): calcd for C_30_H_38_ClN_3_O_3_S [M − H^+^]: 538.22950; found: 538.23074.

*(1R,4aS)-7-Isopropyl-N-(((R)-1-((2-methoxyphenyl)amino)-1-oxopropan-2-yl)carbamothioyl)-1,4a-dimethyl-1,2,3,4,4a,9,10,10a-octahydrophenanthrene-1-carboxamide* (**8f**). Yields 82.38%; ^1^H NMR (400 MHz, CDCl_3_): δ 11.19 (d, *J* = 7.3 Hz, 1H, NH), 8.61 (s, 1H, NH), 8.46 (s, 1H, NH), 8.37 (dd, *J* = 8.0, 1.5 Hz, 1H), 7.15 (d, *J* = 8.2 Hz, 1H), 7.05 (t, *J* = 7.0 Hz, 1H), 7.01 (d, *J* = 9.9 Hz, 1H), 6.95 (d, *J* = 9.0 Hz, 1H), 6.87 (d, *J* = 9.1 Hz, 2H), 5.11 (dd, *J* = 14.1, 7.0 Hz, 1H), 3.87 (s, 3H, OCH_3_), 2.97–2.77 (m, 3H), 2.33 (d, *J* = 13.3 Hz, 1H), 2.08 (d, *J* = 14.3 Hz, 1H), 1.83–1.67 (m, 5H), 1.62 (d, *J* = 7.0 Hz, 3H, CH_3_), 1.53–1.46 (m, 2H), 1.34 (s, 3H, CH_3_), 1.23 (s, 6H, 2 × CH_3_), 1.21 (s, 3H, CH_3_). ^13^C NMR (100 MHz, CDCl_3_): δ 179.4, 179.1, 168.4, 147.8, 146.1, 145.8, 134.1, 127.1, 126.8, 124.0, 123.9, 123.8, 120.9, 119.7, 109.9, 55.6, 55.5, 48.4, 45.0, 37.5, 36.9, 36.8, 33.3, 29.6, 25.0, 23.9, 21.3, 18.3, 17.5, 16.0. HR-MS (*m/z*) (ESI): calcd for C_31_H_41_N_3_O_3_S [M − H^+^]: 534.27903; found: 534.28014.

*(1R,4aS)-7-Isopropyl-N-(((R)-1-((4-methoxyphenyl)amino)-1-oxopropan-2-yl)carbamothioyl)-1,4a-dimethyl-1,2,3,4,4a,9,10,10a-octahydrophenanthrene-1-carboxamide* (**8g**). Yields 81.32%; ^1^H NMR (400 MHz, CDCl_3_): δ 11.13 (d, *J* = 7.4 Hz, 1H, NH), 8.59 (d, *J* = 3.1 Hz, 2H, NH), 7.46 (d, *J* = 9.0 Hz, 2H), 7.15 (d, *J* = 8.2 Hz, 1H), 7.01 (dd, *J* = 8.1, 1.7 Hz, 1H), 6.88 (d, *J* = 1.3 Hz, 1H), 6.84 (d, *J* = 9.0 Hz, 2H), 5.09–5.03 (m, 1H), 3.77 (s, 3H, OCH_3_), 2.94–2.80 (m, 3H), 2.33 (d, *J* = 13.2 Hz, 1H), 2.07 (dd, *J* = 13.0, 2.3 Hz, 1H), 1.80–1.67 (m, 5H), 1.58 (d, *J* = 6.9 Hz, 3H, CH_3_), 1.52–1.46 (m, 2H), 1.33 (s, 3H, CH_3_), 1.23 (s, 6H, 2× CH_3_), 1.21 (s, 3H, CH_3_). ^13^C NMR (100 MHz, CDCl_3_): δ 179.7, 179.3, 168.4, 156.3, 146.1, 145.9, 134.1, 130.7, 126.9, 124.0, 123.9, 121.6, 114.0, 55.4, 55.1, 48.5, 45.1, 37.5, 36.9, 36.9, 33.4, 29.6, 25.1, 23.9, 21.4, 18.4, 16.9, 16.0. HR-MS (*m/z*) (ESI): calcd for C_31_H_41_N_3_O_3_S [M − H^+^]: 534.27903; found: 534.28029.

*(1R,4aS)-N-(((R)-1-((2-Fluorophenyl)amino)-1-oxopropan-2-yl)carbamothioyl)-7-isopropyl-1,4a-dimethyl-1,2,3,4,4a,9,10,10a-octahydrophenanthrene-1-carboxamide* (**8h**). Yields 87.22%; ^1^H NMR (400 MHz, CDCl_3_): δ 11.14 (d, *J* = 7.4 Hz, 1H, NH), 8.65 (s, 1H, NH), 8.59 (s, 1H, NH), 8.30 (s, 1H), 7.15 (d, *J* = 8.2 Hz, 1H), 7.13–7.06 (m, 2H), 7.04 (s, 1H), 7.01 (d, *J* = 8.3 Hz, 1H), 6.88 (s, 1H), 5.16 (dd, *J* = 14.2, 7.1 Hz, 1H), 2.95–2.79 (m, 3H), 2.33 (d, *J* = 13.1 Hz, 1H), 2.07 (d, *J* = 1.7 Hz, 1H), 1.82–1.66 (m, 5H), 1.61 (d, *J* = 7.0 Hz, 3H, CH_3_), 1.51–1.44 (m, 2H), 1.33 (s, 3H, CH_3_), 1.22 (s, 6H, 2 × CH_3_), 1.21 (s, 3H, CH_3_). ^13^C NMR (100MHz, CDCl_3_): δ 179.9, 179.4, 168.8, 153.6, 151.1, 146.0, 145.9, 134.1, 126.8, 125.9, 125.8, 124.6, 124.5, 124.4, 124.3, 123.9, 123.8, 121.7, 121.2, 114.8, 114.6, 55.2, 48.5, 45.0, 37.4, 36.9, 36.8, 33.3, 29.6, 25.0, 23.8, 21.3, 18.3, 16.9, 16.0. HR-MS (*m/z*) (ESI): calcd for C_30_H_38_FN_3_O_2_S [M − H^+^]: 522.25905; found: 522.26047.

*(1R,4aS)-N-(((R)-1-((4-Fluorophenyl)amino)-1-oxopropan-2-yl)carbamothioyl)-7-isopropyl-1,4a-dimethyl-1,2,3,4,4a,9,10,10a-octahydrophenanthrene-1-carboxamide* (**8i**). Yields 81.26%; ^1^H NMR (400 MHz, CDCl_3_): δ 11.15 (d, *J* = 7.4 Hz, 1H, NH), 8.82 (s, 1H, NH), 8.63 (s, 1H, NH), 7.56–7.52 (m, 2H), 7.19 (d, *J* = 8.2 Hz, 1H), 7.06–6.99 (m, 3H), 6.91 (d, *J* = 1.4 Hz, 1H), 5.14–5.07 (m, 1H), 2.96–2.82 (m, 3H), 2.36 (d, *J* = 13.3 Hz, 1H), 2.11 (s, 1H), 1.84–1.66 (m, 5H), 1.61 (d, *J* = 6.9 Hz, 3H, CH_3_), 1.56–1.47 (m, 2H), 1.36 (s, 3H, CH_3_), 1.26 (s, 6H, 2 × CH_3_), 1.24 (s, 3H, CH_3_). ^13^C NMR (100 MHz, CDCl_3_): δ 178.0, 179.5, 168.6, 160.5, 158.1, 146.1, 146.0, 134.1, 133.6, 133.6, 126.9, 124.0, 123.9, 121.7, 121.6, 115.7, 115.4, 55.1, 48.6, 45.1, 37.5, 37.0, 36.9, 33.4, 29.6, 25.1, 23.9, 21.4, 18.4, 16.8, 16.0. HR-MS (*m/z*) (ESI): calcd for C_30_H_38_FN_3_O_2_S [M − H^+^]: 522.25905; found: 522.26019.

*(1R,4aS)-N-(((R)-1-((2-Bromophenyl)amino)-1-oxopropan-2-yl)carbamothioyl)-7-isopropyl-1,4a-dimethyl-1,2,3,4,4a,9,10,10a-octahydrophenanthrene-1-carboxamide* (**8j**). Yields 83.16%; ^1^H NMR (400 MHz, CDCl_3_): δ 11.17 (d, *J* = 7.3 Hz, 1H, NH), 8.69 (s, 1H, NH), 8.41 (s, 1H, NH), 8.35 (d, *J* = 8.3 Hz, 1H), 7.56 (d, *J* = 9.4 Hz, 1H), 7.33 (t, *J* = 8.5 Hz, 1H), 7.18 (d, *J* = 8.2 Hz, 1H), 7.06–6.99 (m, 2H), 6.91 (s, 1H), 5.21–5.15 (m, 1H), 2.96–2.81 (m, 3H), 2.36 (d, *J* = 13.4 Hz, 1H), 2.12 (d, *J* = 12.5 Hz, 1H), 1.85–1.70 (m, 5H), 1.66 (d, *J* = 7.0 Hz, 3H, CH_3_), 1.55–1.48 (m, 2H), 1.37 (s, 3H, CH_3_), 1.25 (s, 6H, 2 × CH_3_), 1.23 (s, 3H, CH_3_). ^13^C NMR (100 MHz, CDCl_3_): δ 180.1, 179.5, 168.8, 146.1, 146.0, 135.2, 134.1, 132.3, 128.2, 126.9, 125.5, 124.0, 123.9, 122.1, 113.7, 55.6, 48.6, 45.2, 37.5, 37.0, 36.9, 33.4, 29.7, 25.1, 23.9, 21.4, 18.4, 17.3, 16.1. HR-MS (*m/z*) (ESI): calcd for C_30_H_38_BrN_3_O_2_S [M + H^+^]: 584.19464; found: 584.19453.

*(1R,4aS)-N-(((R)-1-((4-Bromophenyl)amino)-1-oxopropan-2-yl)carbamothioyl)-7-isopropyl-1,4a-dimethyl-1,2,3,4,4a,9,10,10a-octahydrophenanthrene-1-carboxamide* (**8k**). Yields 82.19%; ^1^H NMR (400 MHz, CDCl_3_): δ 11.10 (d, *J* = 7.5 Hz, 1H, NH), 8.81 (s, 1H, NH), 8.63 (s, 1H, NH), 7.43 (d, *J* = 7.3 Hz, 2H), 7.15 (d, *J* = 8.2 Hz, 1H), 7.01 (d, *J* = 8.2 Hz, 1H), 6.88 (s, 1H), 6.55 (s, 2H), 5.10–5.05 (m, 1H), 2.92–2.78 (m, 3H), 2.33 (d, *J* = 13.3 Hz, 1H), 2.06 (d, *J* = 12.5 Hz, 1H), 1.81–1.65 (m, 5H), 1.57 (d, *J* = 6.9 Hz, 3H, CH3), 1.49–1.42 (m, 2H), 1.32 (s, 3H, CH_3_), 1.22 (d, *J* = 3.1 Hz, 6H, 2 × CH_3_), 1.21 (s, 3H, CH_3_). ^13^C NMR (100 MHz, CDCl_3_): δ 180.1, 179.6, 168.5, 146.1, 145.3, 136.7, 134.1, 131.1, 127.0, 123.9, 121.5, 116.6, 110.1, 55.1, 48.6, 45.1, 37.5, 37.0, 36.9, 33.4, 29.6, 25.1, 23.9, 21.4, 18.4, 16.5, 16.0. HR-MS (*m/z*) (ESI): calcd for C_30_H_38_BrN_3_O_2_S [M + H^+^]: 584.19464; found: 584.19565.

*(1R,4aS)-N-(((R)-1-((3,5-Dimethylphenyl)amino)-1-oxopropan-2-yl)carbamothioyl)-7-isopropyl-1,4a-dimethyl-1,2,3,4,4a,9,10,10a-octahydrophenanthrene-1-carboxamide* (**8l**). Yields 80.25%; ^1^H NMR (400 MHz, CDCl_3_): δ 11.11 (d, *J* = 7.5 Hz, 1H, NH), 8.59 (s, 1H, NH), 8.54 (s, 1H, NH), 7.19 (s, 2H), 7.15 (d, *J* = 8.2 Hz, 1H), 7.00 (d, *J* = 9.6 Hz, 1H), 6.88 (s, 1H), 6.74 (s, 1H), 5.07 (dd, *J* = 13.1, 6.0 Hz, 1H), 2.94–2.78 (m, 3H), 2.33 (d, *J* = 13.2 Hz, 1H), 2.27 (s, 6H, 2 × CH_3_), 2.07 (d, *J* = 14.2 Hz, 1H), 1.83–1.74 (m, 5H), 1.58 (d, *J* = 6.9 Hz, 3H, CH_3_)., 1.52–1.45 (m, 2H), 1.33 (s, 3H, CH_3_), 1.22 (s, 6H, 2 × CH_3_), 1.21 (s, 3H, CH_3_). ^13^C NMR (100 MHz, CDCl_3_): δ 179.5, 179.2, 168.7, 146.0, 145.7, 138.4, 137.3, 134.0, 126.7, 126.0, 123.8, 123.7, 117.6, 55.2, 53.3, 48.4, 44.9, 37.4, 36.8, 36.7, 33.2, 29.5, 24.9, 23.8, 21.1, 17.0, 15.8, 15.7. HR-MS (*m/z*) (ESI): calcd for C_32_H_43_N_3_O_2_S [M − H^+^]: 532.29977; found: 532.30120.

*(1R,4aS)-7-Isopropyl-1,4a-dimethyl-N-(((R)-1-(naphthalen-1-ylamino)-1-oxopropan-2-yl)carbamothioyl)-1,2,3,4,4a,9,10,10a-octahydrophenanthrene-1-carboxamide* (**8m**). Yields 88.15%; ^1^H NMR (400 MHz, CDCl_3_): δ 11.18 (d, *J* = 7.6 Hz, 1H, NH), 8.97 (s, 1H, NH), 8.66 (s, 1H, NH), 8.00 (d, *J* = 7.7 Hz, 2H), 7.82 (d, *J* = 8.5 Hz, 1H), 7.65 (d, *J* = 8.2 Hz, 1H), 7.50 (d, *J* = 6.8 Hz, 1H), 7.47 (d, *J* = 6.2 Hz, 1H), 7.43 (d, *J* = 7.9 Hz, 1H), 7.14 (d, *J* = 8.2 Hz, 1H), 7.01 (d, *J* = 9.8 Hz, 1H), 6.87 (s, 1H), 5.34–5.27 (m, 1H), 2.91–2.79 (m, 3H), 2.32 (d, *J* = 13.0 Hz, 1H), 2.07 (d, *J* = 12.4 Hz, 1H), 1.85–1.69 (m, 5H), 1.66 (d, *J* = 6.9 Hz, 3H, CH_3_), 1.50–1.43 (m, 2H), 1.32 (s, 3H, CH_3_), 1.22 (s, 3H, CH_3_), 1.21 (d, *J* = 1.8 Hz, 6H, 2 × CH_3_). ^13^C NMR (100 MHz, CDCl_3_: δ 179.9, 179.5, 169.2, 146.1, 145.9, 134.1, 133.9, 132.2, 128.5, 126.9, 126.6, 126.2, 125.9, 125.6, 125.5, 124.0, 123.9, 121.1, 120.2, 55.2, 48.5, 45.0, 37.5, 36.9, 36.9, 33.4, 29.6, 25.1, 23.9, 21.4, 18.4, 16.8, 16.0. HR-MS (*m/z*) (ESI): calcd for C_34_H_41_N_3_O_2_S [M − H^+^]: 554.28412; found: 554.28545.

*(1R,4aS)-7-Isopropyl-1,4a-dimethyl-N-(((R)-1-oxo-1-(pyridin-2-ylamino)propan-2-yl)carbamothioyl)-1,2,3,4,4a,9,10,10a-octahydrophenanthrene-1-carboxamide* (**8n**). Yields 81.03%; ^1^H NMR (400 MHz, CDCl_3_): δ 11.16 (s, 1H, NH), 9.37 (s, 1H, NH), 8.63 (s, 1H, NH), 8.35 (s, 1H), 8.23 (d, *J* = 8.2 Hz, 1H), 7.71 (d, *J* = 6.6 Hz, 1H), 7.15 (d, *J* = 8.2 Hz, 1H), 7.08 (d, *J* = 5.1 Hz, 1H), 7.01 (d, *J* = 8.1 Hz, 1H), 6.88 (s, 1H), 5.09 (dd, *J* = 12.2, 5.3 Hz, 1H), 2.94–2.79 (m, 3H), 2.34 (d, *J* = 13.4 Hz, 1H), 2.08 (d, *J* = 10.6 Hz, 1H), 1.88–1.70 (m, 5H), 1.60 (d, *J* = 7.2 Hz, 3H, CH_3_), 1.52–1.46 (m, 2H), 1.34 (s, 3H, CH_3_), 1.23 (d, *J* = 2.7 Hz, 6H, 2 × CH_3_), 1.21 (s, 3H, CH_3_). ^13^C NMR (100 MHz, CDCl_3_): δ 180.1, 179.4, 169.5, 151.1, 147.8, 146.2, 146.0, 138.5, 134.2, 126.9, 124.0, 123.9, 120.1, 114.5, 55.6, 53.4, 48.6, 45.1, 37.6, 37.0, 33.4, 29.6, 25.1, 23.9, 21.4, 18.4, 17.5, 16.1. HR-MS (*m/z*) (ESI): calcd for C_29_H_38_N_4_O_2_S [M − H^+^]: 505.26372; found: 505.26356.

*(1R,4aS)-7-Isopropyl-1,4a-dimethyl-N-(((R)-1-oxo-1-((3,4,5-trimethoxyphenyl)amino)propan-2-yl)carbamothioyl)-1,2,3,4,4a,9,10,10a-octahydrophenanthrene-1-carboxamide* (**8o**). Yields 82.06%; ^1^H NMR (400 MHz, CDCl_3_): δ 11.12 (d, *J* = 7.5 Hz, 1H, NH), 8.72 (s, 1H, NH), 8.59 (s, 1H, NH), 7.16 (d, *J* = 8.2 Hz, 1H), 7.01 (d, *J* = 8.2 Hz, 1H), 6.88 (s, 3H), 5.07 (dd, *J* = 14.2, 7.0 Hz, 1H), 3.84 (s, 6H, 2 × OCH_3_), 3.81 (s, 3H, OCH_3_), 2.94–2.79 (m, 3H), 2.34 (d, *J* = 13.3 Hz, 1H), 2.07 (dd, *J* = 12.5, 1.7 Hz, 1H), 1.85–1.66 (m, 5H), 1.59 (d, *J* = 6.9 Hz, 3H, CH_3_), 1.52–1.45 (m, 2H), 1.34 (s, 3H, CH_3_), 1.23 (s, 6H, 2 × CH_3_), 1.21 (s, 3H, CH_3_).^13^C NMR (100 MHz, CDCl_3_): δ 179.9, 179.5, 168.5, 153.2, 146.1, 146.0, 134.5, 134.1, 133.8, 126.9, 124.0, 123.9, 97.3, 60.9, 56.0, 55.2, 48.6, 45.1, 37.5, 37.0, 36.9, 33.4, 29.6, 25.1, 23.9, 21.4, 18.4, 16.7, 16.0. HR-MS (*m/z*) (ESI): calcd for C_33_H_45_N_3_O_5_S [M − H^+^]: 594.30016; found: 594.30143.

*(1R,4aS)-7-Isopropyl-1,4a-dimethyl-N-(((R)-1-oxo-3-phenyl-1-(phenylamino)propan-2-yl)carbamothioyl)-1,2,3,4,4a,9,10,10a-octahydrophenanthrene-1-carboxamide* (**9a**). Yields 89.23%, ^1^H NMR (400 MHz, CDCl_3_): δ 11.29 (d, *J* = 7.4 Hz, 1H, NH), 8.56 (s, 1H, NH), 7.92 (s, 1H), 7.35 (d, *J* = 7.6 Hz, 2H), 7.30–7.28 (m, 3H), 7.23 (s, 2H), 7.21 (s, 1H), 7.13 (d, *J* = 8.2 Hz, 1H), 7.05 (d, *J* = 7.4 Hz, 1H), 7.00 (d, *J* = 8.2 Hz, 1H), 6.87 (s, 1H), 5.20 (dd, *J* = 14.2, 7.6 Hz, 1H). 3.41–3.35 (m, 1H), 3.24–3.19 (m, 1H), 2.89–2.78 (m, 3H), 2.30 (d, *J* = 13.2 Hz, 1H), 2.01 (d, *J* = 12.5 Hz, 1H), 1.76–1.39 (m, 7H), 1.27 (s, 3H, CH_3_), 1.21 (d, *J* = 6.9 Hz, 6H, 2 × CH_3_), 1.19 (s, 3H, CH_3_). ^13^C NMR (100 MHz, CDCl_3_): δ 179.5, 178.9, 167.4, 146.0, 145.6, 136.9, 135.9, 133.9, 129.2, 128.6, 128.4, 126.9, 126.7, 124.3, 123.8, 123.7, 119.9, 61.2, 48.2, 44.9, 37.4, 37.3, 36.7, 36.5, 33.2, 29.4, 24.8, 23.7, 21.0, 18.2, 15.7. HR-MS (*m/z*) (ESI): calcd for C_36_H_43_N_3_O_2_S [M − H^+^]: 580.29977; found: 580.30102.

*(1R,4aS)-7-Isopropyl-1,4a-dimethyl-N-(((R)-1-oxo-3-phenyl-1-(o-tolylamino)propan-2-yl)carbamothioyl)-1,2,3,4,4a,9,10,10a-octahydrophenanthrene-1-carboxamide* (**9b**). Yields 88.24%, ^1^H NMR (400 MHz, CDCl_3_): δ 11.27 (d, *J* = 7.5 Hz, 1H, NH), 8.59 (s, 1H, NH), 7.87 (d, *J* = 8.0 Hz, 1H), 7.53 (s, 1H), 7.32 (d, *J* = 9.8 Hz, 4H), 7.15 (d, *J* = 8.2 Hz, 2H), 7.10 (d, *J* = 6.8 Hz, 1H), 7.01 (d, *J* = 8.4 Hz, 2H), 6.88 (d, *J* = 1.4 Hz, 1H), 5.26 (dd, *J* = 14.5, 8.1 Hz, 1H), 3.42–3.35 (m, 1H), 3.29–3.22 (m, 1H), 2.90–2.79 (m, 3H), 2.33 (d, *J* = 13.3 Hz, 1H), 2.06 (d, *J* = 12.5 Hz, 1H), 1.98 (s, 3H, CH_3_), 1.75–1.45 (m, 7H), 1.32 (s, 3H, CH_3_), 1.22 (d, *J* = 3.5 Hz, 6H, 2 × CH_3_), 1.21 (s, 3H, CH_3_). ^13^C NMR (100 MHz, CDCl_3_): δ 179.9, 179.1, 167.4, 157.5, 146.1, 145.9, 136.1, 135.2, 134.1, 130.2, 129.2, 128.7, 127.1, 126.8, 126.5, 125.0, 123.8, 122.3, 121.2, 61.3, 48.4, 45.1, 37.5, 37.3, 36.9, 36.7, 33.3, 29.6, 25.0, 23.8, 21.2, 18.3, 17.5, 15.9. HR-MS (*m/z*) (ESI): calcd for C_37_H_45_N_3_O_2_S [M − H^+^]: 618.31302; found: 618.31195.

*(1R,4aS)-7-Isopropyl-1,4a-dimethyl-N-(((R)-1-oxo-3-phenyl-1-(p-tolylamino)propan-2-yl)carbamothioyl)-1,2,3,4,4a,9,10,10a-octahydrophenanthrene-1-carboxamide* (**9c**). Yields 87.14%, ^1^H NMR (400 MHz, CDCl_3_): δ 11.28 (d, *J* = 7.4 Hz, 1H, NH), 8.56 (s, 1H, NH), 7.63 (s, 1H), 7.32 (d, *J* = 4.8 Hz, 4H), 7.23 (d, *J* = 8.5 Hz, 2H), 7.15 (d, *J* = 8.2 Hz, 1H), 7.06 (d, *J* = 8.2 Hz, 2H), 7.01 (dd, *J* = 8.1, 1.7 Hz, 1H), 6.88 (d, *J* = 1.3 Hz, 1H), 5.16 (dd, *J* = 14.3, 7.8 Hz, 1H), 3.42–3.36 (m, 1H), 3.23–3.17 (m, 1H), 2.90–2.79 (m, 3H), 2.28 (s, 3H, CH_3_), 2.03 (d, *J* = 1.9 Hz, 1H), 1.76–1.45 (m, 7H), 1.31 (s, 3H, CH_3_), 1.23 (d, *J* = 4.1 Hz, 6H, 2 × CH_3_), 1.21 (s, 3H, CH_3_). ^13^C NMR (100 MHz, CDCl_3_): δ 179.8, 179.1, 167.2, 146.2, 145.9, 136.3, 136.2, 134.5, 134.1, 129.3, 128.7, 127.1, 126.9, 124.0, 123.9, 120.1, 119.8, 61.4, 48.5, 45.2, 37.6, 37.5, 37.0, 36.8, 33.4, 29.6, 25.0, 23.9, 21.2, 20.8, 18.4, 16.0. HR-MS (*m/z*) (ESI): calcd for C_37_H_45_N_3_O_2_S [M − H^+^]: 594.31542; found: 594.31687.

*(1R,4aS)-N-(((R)-1-((2-Chlorophenyl)amino)-1-oxo-3-phenylpropan-2-yl)carbamothioyl)-7-isopropyl-1,4a-dimethyl-1,2,3,4,4a,9,10,10a-octahydrophenanthrene-1-carboxamide* (**9d**). Yields 82.46%, ^1^H NMR (400 MHz, CDCl_3_): δ 11.29 (d, *J* = 7.4 Hz, 1H, NH), 8.62 (s, 1H, NH), 8.37 (d, *J* = 8.3 Hz, 1H, NH), 8.00 (s, 1H), 7.30 (d, *J* = 6.0 Hz, 4H), 7.27 (d, *J* = 1.4 Hz, 1H), 7.25–7.21 (m, 2H), 7.15 (d, *J* = 8.2 Hz, 1H), 7.03–6.98 (m, 2H), 6.88 (d, *J* = 1.4 Hz, 1H), 5.29 (dd, *J* = 14.2, 7.5 Hz, 1H), 3.43–3.37 (m, 1H), 3.28–3.21 (m, 1H), 2.91–2.79 (m, 3H), 2.33 (d, *J* = 13.1 Hz, 1H), 2.07 (d, *J* = 10.6 Hz, 1H), 1.77–1.39 (m, 7H), 1.32 (s, 3H, CH_3_), 1.22 (d, *J* = 4.0 Hz, 6H, 2 × CH_3_), 1.21 (s, 3H, CH_3_). ^13^C NMR (100 MHz, CDCl_3_): δ 180.0, 179.1, 167.5, 146.1, 145.7, 135.7, 134.0, 133.9, 129.1, 128.8, 128.7, 127.4, 127.1, 126.5, 124.8, 123.9, 123.8, 122.8, 121.5, 61.5, 48.4, 45.1, 37.5, 37.4, 36.8, 36.7, 33.3, 29.5, 24.9, 23.8, 21.2, 18.3, 15.9. HR-MS (*m/z*) (ESI): calcd for C_36_H_42_ClN_3_O_2_S [M + Na^+^]: 638.25840; found: 638.25879.

*(1R,4aS)-N-(((R)-1-((4-Chlorophenyl)amino)-1-oxo-3-phenylpropan-2-yl)carbamothioyl)-7-isopropyl-1,4a-dimethyl-1,2,3,4,4a,9,10,10a-octahydrophenanthrene-1-carboxamide* (**9e**). Yields 85.78%, ^1^H NMR (400 MHz, CDCl_3_): δ 11.27 (d, *J* = 7.4 Hz, 1H, NH), 8.56 (s, 1H, NH), 7.86 (s, 1H), 7.32 (s, 1H), 7.31 (s, 3H), 7.29 (d, *J* = 3.3 Hz, 2H), 7.23 (d, *J* = 8.7 Hz, 2H), 7.15 (d, *J* = 8.2 Hz, 1H), 7.01 (d, *J* = 8.1 Hz, 1H), 6.89 (s, 1H), 5.17 (dd, *J* = 14.7, 7.4 Hz, 1H), 3.42–3.35 (m, 1H), 3.25–3.18 (m, 1H), 2.96–2.76 (m, 3H), 2.34 (d, *J* = 13.1 Hz, 1H), 2.04 (d, *J* = 11.1 Hz, 1H), 1.77–1.42 (m, 7H), 1.31 (s, 3H, CH_3_), 1.23 (d, *J* = 3.6 Hz, 6H, 2 × CH_3_), 1.22 (s, 3H, CH_3_). ^13^C NMR (100 MHz, CDCl_3_): δ 180.2, 179.3, 167.4, 146.2, 146.1, 136.1, 135.7, 134.1, 129.3, 128.9, 128.8, 127.3, 126.9, 124.1, 124.0, 123.9, 121.2, 61.4, 48.6, 45.3, 37.6, 37.3, 37.0, 36.9, 33.5, 29.7, 25.1, 23.9, 21.3, 18.5, 16.0. HR-MS (*m/z*) (ESI): calcd for C_36_H_42_ClN_3_O_2_S [M − H^+^]: 614.26080; found: 614.26064.

*(1R,4aS)-7-Isopropyl-N-(((R)-1-((2-methoxyphenyl)amino)-1-oxo-3-phenylpropan-2-yl)carbamothioyl)-1,4a-dimethyl-1,2,3,4,4a,9,10,10a-octahydrophenanthrene-1-carboxamide* (**9f**). Yields 80.28%, ^1^H NMR (400 MHz, CDCl_3_): δ 11.39 (d, *J* = 7.3 Hz, 1H, NH), 8.64 (s, 1H, NH), 8.41 (d, *J* = 9.3 Hz, 1H, NH), 7.94 (s, 1H), 7.37 (d, *J* = 6.8 Hz, 3H), 7.33 (s, 1H), 7.31 (d, *J* = 3.9 Hz, 1H), 7.20 (d, *J* = 8.2 Hz, 1H), 7.07–7.04 (m, 2H), 6.97 (d, *J* = 7.7 Hz, 1H), 6.93 (s, 1H), 6.82 (d, *J* = 8.1 Hz, 1H), 5.26 (dd, *J* = 13.8, 7.8 Hz, 1H), 3.72 (s, 3H, OCH_3_), 3.52–3.47 (m, 1H), 3.25–3.19 (m, 1H), 2.95–2.84 (m, 3H), 2.38 (d, *J* = 13.0 Hz, 1H), 2.12 (d, *J* = 12.4 Hz, 1H), 1.78–1.49 (m, 7H), 1.38 (s, 3H, CH_3_), 1.28 (d, *J* = 3.0 Hz, 6H, 2 × CH_3_), 1.26 (s, 3H, CH_3_). ^13^C NMR (100 MHz, CDCl_3_): δ 179.5, 178.9, 167.1, 147.7, 146.1, 145.8, 136.1, 134.1, 129.2, 128.5, 126.9, 126.8, 123.9, 123.8, 120.9, 120.7, 120.7, 119.6, 109.8, 61.7, 55.4, 48.4, 45.1, 37.8, 37.5, 36.9, 36.8, 33.3, 29.5, 24.9, 23.8, 21.2, 18.3, 15.9. HR-MS (*m/z*) (ESI): calcd for C_36_H_45_N_3_O_3_S [M − H^+^]: 610.31033; found: 610.31051.

*(1R,4aS)-7-Isopropyl-N-(((R)-1-((4-methoxyphenyl)amino)-1-oxo-3-phenylpropan-2-yl)carbamothioyl)-1,4a-dimethyl-1,2,3,4,4a,9,10,10a-octahydrophenanthrene-1-carboxamide* (**9g**). Yields 81.57%, ^1^H NMR (400 MHz, CDCl_3_): δ 11.29 (d, *J* = 7.4 Hz, 1H, NH), 8.55 (s, 1H, NH), 7.54 (s, 1H), 7.33 (d, *J* = 4.4 Hz, 4H), 7.26 (s, 1H), 7.24 (s, 1H), 7.16 (d, *J* = 8.2 Hz, 1H), 7.01 (d, *J* = 6.5 Hz, 1H), 6.89 (s, 1H), 6.80 (d, *J* = 9.0 Hz, 2H), 5.14 (dd, *J* = 14.4, 7.8 Hz, 1H), 3.77 (s, 3H, OCH_3_), 3.42–3.38 (m, 1H), 3.23–3.17 (m, 1H), 2.89–2.79 (m, 3H), 2.33 (d, *J* = 13.1 Hz, 1H), 2.05 (d, *J* = 12.4 Hz, 1H), 1.73–1.43 (m, 7H), 1.32 (s, 3H, CH_3_), 1.23 (d, *J* = 2.8 Hz, 6H, 2 × CH_3_), 1.22 (s, 3H, CH_3_). ^13^C NMR (100 MHz, CDCl_3_): δ 179.8, 179.2, 167.1, 156.5, 146.2, 146.0, 136.3, 134.2, 130.1, 129.4, 128.8, 127.2, 126.9, 124.0, 123.9, 121.8, 114.0, 61.5, 55.4, 48.6, 45.2, 37.6, 37.5, 37.0, 36.9, 33.4, 29.6, 25.1, 23.9, 21.3, 18.4, 16.04. HR-MS (*m/z*) (ESI): calcd for C_36_H_45_N_3_O_3_S [M + Na^+^]: 634.30793; found: 634.30579.

*(1R,4aS)-N-(((R)-1-((2-Fluorophenyl)amino)-1-oxo-3-phenylpropan-2-yl)carbamothioyl)-7-isopropyl-1,4a-dimethyl-1,2,3,4,4a,9,10,10a-octahydrophenanthrene-1-carboxamide* (**9h**). Yields 85.27%, ^1^H NMR (400 MHz, CDCl_3_): δ 11.30 (d, *J* = 9.9 Hz, 1H, NH), 8.60 (s, 1H, NH), 8.26 (s, 1H, NH), 7.91 (dd, *J* = 13.0, 2.3 Hz, 1H), 7.30 (s, 3H), 7.28–7.21 (m, 2H), 7.15 (d, *J* = 8.2 Hz, 1H), 7.11–7.05 (m, 1H), 7.01 (d, *J* = 4.4 Hz, 2H), 6.99 (d, *J* = 1.6 Hz, 1H), 6.88 (d, *J* = 5.4 Hz, 1H), 5.26 (dd, *J* = 12.6, 5.2 Hz, 1H), 3.42–3.35(m, 1H), 3.28–3.22 (m, 1H), 2.92–2.79 (m, 3H), 2.32 (d, *J* = 13.3 Hz, 1H), 2.09–2.02 (m, 1H), 1.76–1.41 (m, 7H), 1.31 (s, 3H, CH_3_), 1.23 (d, *J* = 2.5 Hz, 3H, CH_3_), 1.21 (d, *J* = 2.2 Hz, 6H, 2 × CH_3_).^13^C NMR (100MHz, CDCl_3_): δ 179.9, 179.0, 167.6, 153.2, 151.3, 146.1, 145.7, 135.7, 134.0, 129.1, 128.5, 127.0, 126.7, 125.5, 125.4, 124.6, 124.5, 124.2, 124.1, 123.8, 123.7, 123.6, 121.8, 114.7, 114.5, 61.2, 48.3, 45.0, 37.4, 37.3, 36.8, 36.6, 33.2, 29.5, 24.9, 23.8, 21.1, 18.3, 15.8. HR-MS (*m/z*) (ESI): calcd for C_36_H_42_FN_3_O_2_S [M − H^+^]: 598.29035; found: 598.29175.

*(1R,4aS)-N-(((R)-1-((4-Fluorophenyl)amino)-1-oxo-3-phenylpropan-2-yl)carbamothioyl)-7-isopropyl-1,4a-dimethyl-1,2,3,4,4a,9,10,10a-octahydrophenanthrene-1-carboxamide* (**9i**). Yields 80.13%, ^1^H NMR (400 MHz, CDCl_3_): δ 11.35 (d, *J* = 7.3 Hz, 1H, NH). 8.63 (s, 1H, NH), 8.03 (s, 1H, NH), 7.38 (d, *J* = 4.8 Hz, 1H), 7.37 (s, 3H), 7.32 (dd, *J* = 9.3, 4.1 Hz, 2H), 7.21 (d, *J* = 8.2 Hz, 1H), 7.08 (d, *J* = 9.7 Hz, 1H), 7.00 (d, *J* = 8.6 Hz, 2H), 6.96 (s, 1H), 6.94 (s, 1H), 5.25 (dd, *J* = 14.5, 7.4 Hz, 1H), 3.55–3.38 (m, 1H), 3.33–3.23 (m, 1H), 2.95–2.87 (m, 3H), 2.38 (d, *J* = 13.2 Hz, 1H), 2.08 (d, *J* = 12.5 Hz, 1H), 1.80–1.47 (m, 7H), 1.35 (s, 3H, CH_3_), 1.29 (d, *J* = 6.9 Hz, 6H, 2 × CH_3_), 1.27 (s, 3H, CH_3_).^13^C NMR (100 MHz, CDCl_3_): δ 179.7, 179.0, 167.5, 160.2, 158.3, 146.0, 145.7, 135.9, 133.9, 132.9, 129.1, 128.5, 127.0, 126.7, 123.9, 123.8, 121.8, 115.4, 115.2, 61.2, 48.4, 45.1, 37.4, 36.8, 36.8, 36.6, 33.3, 29.5, 24.9, 23.7, 21.1, 18.3, 15.8. HR-MS (*m/z*) (ESI): calcd for C_36_H_42_FN_3_O_2_S [M − H^+^]: 598.29035; found: 598.29118.

*(1R,4aS)-N-(((R)-1-((2-Bromophenyl)amino)-1-oxo-3-phenylpropan-2-yl)carbamothioyl)-7-isopropyl-1,4a-dimethyl-1,2,3,4,4a,9,10,10a-octahydrophenanthrene-1-carboxamide* (**9j**). Yields 88.33%, ^1^H NMR (400 MHz, CDCl_3_): δ 11.21 (d, *J* = 7.4 Hz, 1H, NH), 8.54 (s, 1H, NH), 8.28 (d, *J* = 8.3 Hz, 1H, NH), 7.91 (s, 1H), 7.21 (d, *J* = 6.0 Hz, 4H), 7.19 (d, *J* = 1.4 Hz, 1H), 7.17–7.12 (m, 2H), 7.07 (d, *J* = 8.2 Hz, 1H), 6.95–6.90 (m, 2H), 6.80 (d, *J* = 1.4 Hz, 1H), 5.21 (dd, *J* = 14.2, 7.5 Hz, 1H), 3.35–3.28 (m, 1H), 3.20–3.13 (m, 1H), 2.83–2.71 (m, 3H), 2.24 (d, *J* = 13.1 Hz, 1H), 1.99 (d, *J* = 10.6 Hz, 1H), 1.69–1.30 (m, 7H), 1.24 (s, 3H, CH_3_), 1.14 (d, *J* = 4.0 Hz, 6H, 2 × CH_3_), 1.13 (s, 3H, CH_3_). ^13^C NMR (100 MHz, CDCl_3_): δ 180.0, 179.2, 167.5, 146.1, 145.8, 135.7, 134.0, 133.9, 129.1, 128.8, 128.7, 127.4, 127.1, 126.8, 124.8, 123.9, 123.8, 122.8, 121.5, 61.5, 48.4, 45.1, 37.5, 37.4, 36.9, 36.7, 33.3, 29.6, 24.9, 23.8, 21.2, 18.3, 16.0. HR-MS (*m/z*) (ESI): calcd for C_36_H_42_BrN_3_O_2_S [M + H^+^]: 660.22594; found: 660.22639.

*(1R,4aS)-N-(((R)-1-((4-Bromophenyl)amino)-1-oxo-3-phenylpropan-2-yl)carbamothioyl)-7-isopropyl-1,4a-dimethyl-1,2,3,4,4a,9,10,10a-octahydrophenanthrene-1-carboxamide* (**9k**). Yields 82.34%, ^1^H NMR (400 MHz, CDCl_3_): δ 11.32 (d, *J* = 7.4 Hz, 1H, NH), 8.60 (s, 1H, NH), 7.98 (s, 1H, NH), 7.40 (d, *J* = 8.8 Hz, 2H), 7.34 (d, *J* = 3.6 Hz, 4H), 7.33–7.31 (m, 1H), 7.29 (d, *J* = 8.9 Hz, 2H), 7.19 (d, *J* = 8.2 Hz, 1H), 7.05 (d, *J* = 8.2 Hz, 1H), 6.92 (s, 1H), 5.22 (dd, *J* = 14.6, 7.4 Hz, 1H), 3.45–3.39 (m, 1H), 3.28–3.21 (m, 1H), 2.94–2.83 (m, 3H), 2.37 (d, *J* = 13.1 Hz, 1H), 2.06 (d, *J* = 14.1 Hz, 1H), 1.82–1.44 (m, 7H), 1.34 (s, 3H, CH_3_), 1.27 (s, 3H, CH_3_), 1.26 (s, 6H, 2 × CH_3_). ^13^C NMR (100 MHz, CDCl_3_): δ 179.9, 179.2, 167.5, 146.1, 145.9,136.1, 136.0, 134.1, 131.8, 129.3, 128.7, 127.2, 126.8, 124.0, 123.9, 121.5, 117.1, 61.4, 48.5, 45.1, 37.5, 37.4, 36.9, 36.8, 33.4, 29.6, 25.0, 23.9, 21.2, 18.4, 15.9. HR-MS (*m/z*) (ESI): calcd for C_36_H_42_BrN_3_O_2_S [M + H^+^]: 660.22594; found: 660.22566.

*(1R,4aS)-N-(((R)-1-((3,5-Dimethylphenyl)amino)-1-oxo-3-phenylpropan-2-yl)carbamothioyl)-7-isopropyl-1,4a-dimethyl-1,2,3,4,4a,9,10,10a-octahydrophenanthrene-1-carboxamide* (**9l**). Yields 82.57%, ^1^H NMR (400 MHz, CDCl_3_): δ 11.34 (d, *J* = 7.4 Hz, 1H, NH), 8.62 (s, 1H, NH), 7.76 (s, 1H), 7.37 (d, *J* = 4.6 Hz, 4H), 7.20 (d, *J* = 8.2 Hz, 1H), 7.05 (s, 3H), 6.94 (s, 1H), 6.78 (s, 1H), 5.23 (dd, *J* = 14.4, 7.5 Hz, 1H), 3.47–3.41 (m, 1H), 3.31–3.25 (m, 1H), 2.94–2.84 (m, 3H), 2.30 (s, 6H, 2 × CH_3_), 1.82–1.51 (m, 7H), 1.36 (s, 3H, CH_3_), 1.29 (s, 3H, CH_3_), 1.27 (s, 6H, 2 × CH_3_). ^13^C NMR (100 MHz, CDCl_3_): δ 179.9, 179.2, 167.2, 146.2, 146.0, 138.6, 136.9, 136.3, 134.2, 129.3, 128.8, 127.1, 126.9, 126.3, 124.0, 123.9, 117.7, 61.5, 48.5, 45.2, 37.6, 37.4, 37.0, 36.9, 33.4, 29.6, 25.0, 23.9, 21.3, 21.2, 18.4, 16.0. HR-MS (*m/z*) (ESI): calcd for C_38_H_47_N_3_O_2_S [M − H^+^]: 608.33107; found: 608.33232.

*(1R,4aS)-7-Isopropyl-1,4a-dimethyl-N-(((R)-1-(naphthalen-1-ylamino)-1-oxo-3-phenylpropan-2-yl)carbamothioyl)-1,2,3,4,4a,9,10,10a-octahydrophenanthrene-1-carboxamide* (**9m**). Yields 84.09%, ^1^H NMR (400 MHz, CDCl_3_): δ 11.34 (d, *J* = 7.5 Hz, 1H, NH), 8.62 (s, 1H, NH), 8.18 (s, 1H, NH), 7.94 (d, *J* = 7.5 Hz, 1H), 7.79 (d, *J* = 8.0 Hz, 1H), 7.64 (d, *J* = 8.2 Hz, 1H), 7.42 (d, *J* = 8.0 Hz, 2H), 7.38 (d, *J* = 5.8 Hz, 4H), 7.35–7.30 (m, 3H), 7.15 (d, *J* = 8.2 Hz, 1H), 7.01 (d, *J* = 8.0 Hz, 1H), 6.88 (s, 1H), 5.42 (dd, *J* = 14.6, 7.4 Hz, 1H), 3.43 (d, *J* = 6.4 Hz, 1H), 3.35–3.28 (m, 1H), 2.89–2.78 (m, 3H), 2.32 (d, *J* = 13.2 Hz, 1H), 2.07 (s, 1H), 1.75–1.43 (m, 7H), 1.30 (s, 3H, CH_3_), 1.23 (s, 3H, CH_3_), 1.21 (s, 6H, 2 × CH_3_). ^13^C NMR (100 MHz, CDCl_3_): δ 180.2, 179.3, 168.0, 146.2, 146.1, 136.3, 134.2, 133.9, 131.8, 129.4, 129.0, 128.5, 127.2, 126.9, 126.5, 126.2, 125.9, 125.7, 125.6, 124.1, 123.9, 120.9, 120.1, 61.6, 48.6, 45.2, 37.6, 37.5, 37.0, 36.9, 33.4, 29.7, 25.1, 23.9, 21.3, 18.5, 16.1. HR-MS (*m/z*) (ESI): calcd for C_40_H_45_N_4_O_2_S [M − H^+^]: 630.31542; found: 630.31666.

*(1R,4aS)-7-Isopropyl-1,4a-dimethyl-N-(((R)-1-oxo-3-phenyl-1-(pyridin-2-ylamino)propan-2-yl)carbamothioyl)-1,2,3,4,4a,9,10,10a-octahydrophenanthrene-1-carboxamide* (**9n**). Yields 88.56%, ^1^H NMR (400 MHz, CDCl_3_): δ 11.19 (d, *J* = 7.1 Hz, 1H, NH), 9.09 (s, 1H, NH), 8.57 (s, 1H), 8.23 (d, *J* = 6.3 Hz, 2H), 7.71 (s, 1H), 7.25 (d, *J* = 3.7 Hz, 4H), 7.15 (d, *J* = 8.2 Hz, 1H), 7.06 (d, *J* = 5.9 Hz, 1H), 7.01 (d, *J* = 8.2 Hz, 1H), 6.89 (s, 1H), 5.23 (dd, *J* = 14.3, 7.8 Hz, 1H), 3.35–3.26 (m, 2H), 2.90–2.80 (m, 3H), 2.33 (d, *J* = 13.3 Hz, 1H), 2.05 (d, *J* = 12.5 Hz, 1H), 1.76–1.49 (m, 7H), 1.31 (s, 3H, CH_3_), 1.23 (d, *J* = 3.5 Hz, 6H, 2 × CH_3_), 1.22 (s, 3H, CH_3_). ^13^C NMR (100 MHz, CDCl_3_): δ 180.4, 178.9, 168.3, 150.8, 147.5, 146.2, 146.0, 138.5, 135.7, 134.2, 129.3, 128.6, 127.2, 126.9, 124.0, 123.9, 120.0, 114.5, 61.6, 48.5, 45.1, 37.7, 37.6, 37.0, 36.9, 33.4, 29.6, 25.0, 23.9, 21.3, 18.4, 16.0. HR-MS (*m/z*) (ESI): calcd for C_37_H_42_N_4_O_2_S [M + H^+^]: 583.31067; found: 583.3098.

*(1R,4aS)-7-Isopropyl-1,4a-dimethyl-N-(((R)-1-oxo-3-phenyl-1-((3,4,5-trimethoxyphenyl)amino)propan-2-yl)carbamothioyl)-1,2,3,4,4a,9,10,10a-octahydrophenanthrene-1-carboxamide* (**9o**). Yields 85.32%, ^1^H NMR (400 MHz, CDCl_3_): δ 11.32 (d, *J* = 7.3 Hz, 1H, NH), 8.59 (s, 1H, NH), 7.80 (s, 1H), 7.34 (d, *J* = 4.4 Hz, 4H), 7.17 (d, *J* = 8.2 Hz, 1H), 7.03 (d, *J* = 8.2 Hz, 1H), 6.90 (s, 1H), 6.67 (s, 2H), 5.19 (dd, *J* = 14.5, 7.4 Hz, 1H), 3.81 (s, 12H, 3 × OCH_3_), 3.45–3.40 (m, 1H), 3.27–3.21 (m, 1H), 2.92–2.81 (m, 3H), 2.35 (d, *J* = 13.2 Hz, 1H), 2.06 (d, *J* = 10.8 Hz, 1H), 1.78–1.48 (m, 7H), 1.33 (s, 3H, CH_3_), 1.24 (d, *J* = 4.6 Hz, 6H, 2 × CH_3_), 1.23 (s, 3H, CH_3_). ^13^C NMR (100 MHz, CDCl_3_): δ 179.8, 179.2, 167.3, 153.0, 146.0, 145.9, 136.1, 134.6, 134.0, 133.1, 129.3, 128.7, 127.0, 126.8, 124.0, 123.8, 97.4, 61.4, 60.8, 55.9, 48.5, 45.1, 37.5, 37.4, 36.9, 36.8, 33.3, 29.5, 24.9, 23.8, 21.2, 18.3, 15.9. HR-MS (*m/z*) (ESI): calcd for C_39_H_49_N_3_O_5_S [M − H^+^]: 670.33146; found: 670.33304.

### 3.2. Biological Assays

#### 3.2.1. Cytotoxicity of Rhein Derivatives

General procedure for cytotoxic evaluation *in vitro:* HeLa, HepG2, SK-OV-3 and MGC-803 cells were seeded into 96-well microculture plates and allowed to adhere for 24 h, respectively. After cells were exposed to compounds at concentrations from 100 to 0.01 μM for 48 h, medium was aspirated and replenished with complete medium. IC_50_ was evaluated by MTT tetrazolium dye assay. Each experiment was performed three times; Statistical analysis: All statistical analysis was performed with SPSS Version 10 (SPSS, Chicago, IL, USA). Data was analyzed by one-way ANOVA. Mean separations were performed using the least significant difference method. Each experiment was replicated thrice, and all experiments yielded similar results. Measurements from all the replicates were combined, and treatment effects were analyzed.

#### 3.2.2. Apoptosis Assessment by AO/EB Staining

Cells were seeded at a concentration of 5 × 104 cell/ mL in a volume of 2 mL on a sterile cover slip in six-well tissue culture plates. Following incubation, the medium was removed and replaced with fresh medium plus 10% fetal bovine serum and supplemented with compound **9n** (15 μM). After the treatment period, the cover slip with monolayer cells was inverted on a glass slide with 20 μL of AO/EB stain (100 mg/mL). Fluorescence was read on a slide with 20 μL of AO/EB stain (100 mg/mL) using a Nikon ECLIPSETE2000-S fluorescence microscope (Olympus, Tokyo, Japan).

#### 3.2.3. Hoechst 33258 Staining

General procedure for Hoechst 33258 staining: cells grown on a sterile cover slip in six-well tissue culture plates were treated with compounds for a certain range of time. The culture medium containing compounds was removed, and the cells were fixed in 4% paraformaldehyde for 10 min. After being washed twice with PBS, the cells were stained with 0.5 mL of Hoechst 33258 (Beyotime, Jiangsu, China) for 5 min and then again washed twice with PBS. The stained nuclei were observed under a Nikon ECLIPSETE2000-S fluorescence microscope using 350 nm excitation and 460 nm emission.

#### 3.2.4. JC-1 Staining

General procedure for mitochondrial membrane potential staining: JC-1 probe was employed to measure mitochondrial depolarization in NCI-H460 cells. Briefly, cells cultured in six-well plates after indicated treatments were incubated with an equal volume of JC-1 staining solution (5 μg/mL) at 37 °C for 20 min and rinsed twice with PBS. Mitochondrial membrane potentials were monitored by determining the relative amounts of dual emissions from mitochondrial JC-1 monomers or aggregates using an Nikon ECLIPSETE2000-S fluorescent microscope. Mitochondrial depolarization is indicated by an increase in the green/red fluorescence intensity ratio.

#### 3.2.5. TUNEL Assay

The TUNEL method was performed to label the 3′-end of fragmented DNA of the apoptotic HeLa cells. The cells treated as indicated were fixed with 4% paraformaldehyde phosphate buffer saline, rinsed with PBS, then permeabilized by 0.1% Triton X-100 for FITC end-labeling the fragmented DNA of the apoptotic HeLa cells using TUNEL cell (Beyotime, Jiangsu, China) apoptosis detection kit. The FITC-labeled TUNEL-positive cells were imaged under a fluorescent microscopy by using 488 nm excitation and 530 nm emission.

#### 3.2.6. Apoptosis Study by Flow Cytometry Assay

General procedure apoptosis ratios: Prepared HeLa cells (1 × 106 cells/mL) were washed twice with cold PBS and then re-suspended gently in 500 μL of binding buffer. Thereafter, cells were stained in 5 μL of Annexin V-FITC and shaken well. Finally, the cells were mixed with 5 μL of PI, incubated for 20 min in the dark and subsequently analyzed using FACSCalibur (Becton Dickinson, Franklin Lakes, NJ, USA).

#### 3.2.7. Investigation of Cell Cycle Distribution

General procedure cell cycle: HeLa cells were maintained in Dulbecco’s modified Eagle’s medium with 10% fetal calf serum in 5% CO_2_ at 37 °C. Cells were harvested by trypsinization and rinsed with PBS. After centrifugation, the pellet (10^5^–10^6^ cells) was suspended in 1 mL of PBS and kept on ice for 5 min. The cell suspension was then fixed by the drop-wise addition of 9 mL precooled (4 °C) 75% ethanol with violent shaking. Fixed samples were kept at 4 °C until use. For staining, cells were centrifuged, resuspended in PBS, digested with 500 μL of RNase A (250 mg/mL), and treated with 25 μL of propidium iodide (PI) (0.15 mM), then incubated for 30 min at 4 °C. PI-positive cells were counted with a FACScan Fluorescence-activated cell sorter (FACS). The population of cells in each cell-cycle phase was determined using Cell Modi FIT software (Becton Dickinson).

### 3.3. Statistics

The data were processed by the Student’s *t*-test with the significance level *p* ≤ 0.05 using SPSS.

## 4. Conclusions

In this study, a series of novel DHAA acyl-thiourea derivatives were designed and synthesized, and their cell growth inhibition activities against the HeLa, SK-OV-3 and MGC-803 cell lines and the HL-7702 normal human river cell line were evaluated using MTT assay. The *in vitro* antitumor activity screening revealed that some compounds exhibited better inhibition activities than the commercial anticancer drug 5-FU. The apoptosis-inducing activity of representative compound **9n** in HeLa cells were investigated by acridine orange/ethidium bromide staining, Hoechst 33258 staining, JC-1 mitochondrial membrane potential staining, TUNEL assay and flow cytometry. Cell cycle analysis indicated that compound **9n** could arrest the HeLa cell line in S stage. Furthermore, molecular mechanism studies suggested that compound **9n** was found to induce apoptosis in HeLa cells via the mitochondrial pathway, including an increase of the production of ROS and intracellular Ca^2+^, loss of mitochondrial membrane potential and activation of caspase-3. The above results demonstrate that the rational design of DHAA acyl derivatives as novel antitumor leads is feasible. Investigations into the precise mechanisms of action is currently under way.
